# The role of mitochondria in immune-mediated disease: the dangers of a split personality

**DOI:** 10.1186/s13075-016-1063-5

**Published:** 2016-07-16

**Authors:** David S. Pisetsky

**Affiliations:** Duke University Medical Center, and Medical Research Service, VA Medical Center, Durham, NC 27710 USA

Each cell in the body is endowed with a rich and diverse set of organelles that perform the specialized activities essential for cell survival and differentiated cell function. These organelles can be defined by morphology as well as biochemical composition, although the critical distinguishing feature is function. The exact number of these organelles differs among cell types. Indeed, the appearance of cells is, by and large, determined by the number of organelles and their disposition within the confines of the cell.

In the study of the cell, our colleagues in cell biology and molecular biology have been very practical and narrowly and precisely focused on the intracellular function of organelles. In this utilitarian conceptualization, an organelle does not have a personality. It has a job. Immunologists are a different breed, however, and go beyond the intracellular space in analyzing the role of molecules and structures. Importantly, immunologists know that these entities can have a complex and shifting identity that transcends the usual intracellular function.

As shown by seminal experiments stemming from the enunciation of the danger hypothesis, it is now clear that, depending on their location, molecules and organelles can have very distinct personalities. These personalities are often split and sometimes multiple. While inside the cell these structures have one function, outside the cell, they can have another—often immunologic. HMGB1 is the now classic example of this phenomenon. A non-histone nuclear protein, HMGB1 mediates chromosomal architecture and transcriptional regulation inside the nucleus; when it gets outside the cell during cell death or activation it can stimulate inflammation.

When it comes to organelles with dynamic personalities, the mitochondrion is at the top of the list. These structures overflow with personality. Mitochondria are key to energy production but they are also a storehouse of molecules to mediate cell death by apoptosis. Mitochondria can change shape and number dramatically and are also prone to breakdown and disruption. Furthermore, after millions and billions of years of evolution, mitochondria have a serious identity problem. Are they prokaryotes enjoying a cozy and symbiotic residence in eukaryotic cells? Or are they eukaryotic organelles that have retained prokaryotic features to allow better energy generation?

Mitochondria have long been viewed as endosymbionts which may have evolved from alpha-proteobacteria. The evolution has not been complete, however, and this situation leads to an important role in immunology. As has now been shown in both in vivo and in vitro systems, mitochondria have powerful immune activities that relate to the molecular properties of key components that resemble their bacterial counterparts. Thus, mitochondria have their own DNA, a 16.5 K closed circle that differs from mammalian DNA in the status of the CpG dinucleotides. Like bacterial DNA, mitochondrial DNA contains unmethylated CpG motifs that allow stimulation of Toll-like (TLR) 9 receptor and likely other internal nucleic acid sensors as well.

Boosting the immune activity of mitochondrial DNA is the presence of 8-oxo-guanine residues. These residues arise from oxidative damage by reactive oxygen species (ROS) and can occur in mitochondrial DNA much more frequently than nuclear DNA. The proclivity for these modifications may result from the proximity of mitochondrial DNA to the electron transport system in the interstices of the mitochondria. Furthermore, since mitochondria DNA is not protected by histones, damage from ROS may occur more readily.

Two other biochemical features may contribute to immune potential. In mitochondria, protein synthesis starts with a formylated amino acid just as in the case of bacteria. Formylated peptides can stimulate receptors on neutrophils to induce activation and chemotaxis. Another mitochondrial protein called TFAM is also immunologically active. TFAM is structurally similar to HMGB1 and, like its counterpart in the nucleus, TFAM can serve as an alarmin and drive inflammation.

With cell stress, certain molecules can leave the mitochondria as permeability breaks down. Cytochrome c can leave the mitochondrial sinking ship and interact with other molecules to drive apoptosis. Similarly, mitochondrial DNA, once outside the confines of the mitochondria, can interact with internal sensors to activate the inflammasome. With more severe cell dysfunction, these mitochondrial products all leave the cell when the cell dies.

In addition to leakage or release of individual components, whole mitochondria can exit the cell to carry their cargo of dangerous molecules. With stimulation of eosinophils, exteriorization of whole mitochondria can occur by a nifty mechanism called the mitochondrial catapult. Another setting in which whole mitochondria are present in an extracellular location is platelet activation. Among microparticles following platelet activation are whole mitochondria as well as microparticles harboring mitochondria inside.

The presence of whole mitochondria in the extracellular space is remarkable, suggesting similarity to that which occurs with bacteremia during an infection. This situation has led to speculation that mitochondria are important drivers of sterile inflammation and conditions such as shock. In chronic autoimmune and inflammatory disease, mitochondrial products can act systemically as well as locally and contribute to the pathogenesis of conditions like rheumatoid arthritis, where mitochondrial DNA in the joint can promote synovitis.

In view of the shifting personality of mitochondria and their tendency to be downright dangerous, terminology is evolving. On one hand, mitochondrial products are DAMPs (damage-associated molecular patterns), since their activity becomes manifest with cell death and damage. On the other hand, these products can be considered PAMPs (pathogen-associated molecular patterns) since their activities derive from some of the same molecular structures as bacterial products. To avoid conflict and endless argument, a more neutral and non-committal term may work: MAMP or mitochondrial-associated molecular pattern.

The recognition of the mitochondrion’s immune power adds another dimension to the emerging picture of the repurposing of molecules to drive innate immunity. While an understanding of the role of mitochondria in immune signaling is just beginning, the message is clear. Mitochondria are unique in their biological split personality. Future studies on autoimmune and inflammatory disease will have to determine which aspect of this split is a source of health and which a source of disorder.

## Abbreviation

ROS, reactive oxygen species
